# Supplementary material for: Are carbon emissions trading and green financial instruments synergistic? -Comprehensive quantitative research based on content analysis
Source: PLoS One. 2024 Mar 7;19(3):e0298601. doi: 10.1371/journal.pone.0298601 (PMC10919601; doi:10.1371/journal.pone.0298601)
Supplement: S1 Data — (ZIP) [file pone.0298601.s001.zip › Data/地级市Z_F工作报告.zip!/║┌┴·╜¡/║┌║╙╩╨/└┤╫╘╣1⁄2╓┌║┼í╛┬φ┐╦╩2╛▌═°í┐_2008.docx]

各位代表：

现在，我代表市人民政府向大会作工作报告，请予审议，并请列席会议的同志提出意见。

一、2007年工作回顾

过去的一年，政府工作在省委、省政府和市委的正确领导下，高举邓小平理论和“三个代表”重要思想伟大旗帜，坚持科学发展观，深入贯彻落实省十次党代会和市四次党代会精神，紧紧围绕市四届人大一次会议确定的目标，努力克服诸多困难和不利因素，求实开拓，较好地完成了各项任务。

——经济持续快速健康发展。预计全市生产总值实现159亿元，增长11.4%，连续四年保持两位数增长，其中，一二三次产业分别增长8.7%、15%和12%。城镇固定资产投资39亿元，增长28%，是近十年增幅最高的一年。全市全口径财政收入9.04亿元，一般预算收入4.7亿元，分别增长28.8%和30.6%。市直全口径财政收入2.86亿元，一般预算收入1.3亿元，分别增长27.8%和29.2%。社会消费品零售总额33亿元，增长15%。城镇居民人均可支配收入6767元，农民人均纯收入3948元，分别增长20%和12.7%。万元地区生产总值能耗下降4%。主要经济指标持续增长，经济运行呈现速度快、效益好、后劲足的良好势头。

——工业速度效益同步提升。预计全市规模以上工业增加值实现8.5亿元，增长18%。地方规模以上工业增加值实现6.9亿元，增长20%；利税2.2亿元，增长18%。原煤产量380万吨，工业硅产量3.5万吨，俄电加工区耗电5亿度。电力、煤炭、建材、冶金等重点行业的拉动作用增强，对工业增长贡献率达到89.5%。国企改革继续推进，西岗子煤矿重组改制、西沟电站国有股减持和资本重组顺利完成，北安庆华厂并轨工作进展顺利。运行监控进一步加强，重点企业生产得到有效保障。

——农村经济保持平稳增长。积极应对50年一遇的严重旱灾，农业增加值预计实现36亿元，增长2%。完成播种面积1250万亩，粮豆薯总产152万吨。种植结构不断优化，小麦和杂粮杂豆播种面积分别增长45%和31%，绿色有机食品监测面积350万亩，增长34.6%。畜牧业增加值预计实现5亿元，增长8%。场县共建更加深入，农机跨区作业382万亩，基础设施、社会事业等方面合作日益密切。组建农机合作社55个，田间综合机械化程度达到93%。林权改革试点在全省率先启动。转移农村劳动力16.8万人。开发境外土地70万亩，增长40%。新农村建设进展顺利，省级试点村达到75个，落实资金1740万元，村级事业发展基金试点工作启动实施，基础设施和社会事业进一步加强。建成农村公路1617公里，完成计划的168%，建设里程为历年之最。解决了1.6万人饮水安全问题。

——对外开放水平不断提高。“两国一城”建设扎实推进，“中俄国际风情节”成功举办，落实双方政府会谈纪要事项70项，43个部门与俄方建立了沟通机制，地区间合作进一步深入。对外贸易进出口总值预计实现22亿美元，增长39%。在俄境内木材采伐50万立方米，承包建筑工程20万平方米，对俄蔬菜出口基地达到8万亩。促成省电力公司收购星河电力公司，扩大了俄电加工区范围。阿州—黑河油品储运、24万吨磷酸二铵等合作项目积极推进。黑河港改扩建一期工程竣工，口岸出入境人数126.9万人次，过货量38.6 万吨，分别增长16.2%和32%。招商引资规模和质量实现新突破，共落实项目193个，其中亿元以上项目17个；实际到位资金28.3亿元，增长49%。与阿州布市赴“哈洽会”和山东、福建联合招商取得实效。接待国内外旅游者125万人次，其中边境游42.9万人次，分别增长11.6%和15%；旅游总收入16.2亿元，增长14%。中俄民族风情园鄂伦春区基本建成。五大连池景区成功跻身国家4A级景区，并荣膺“中国最具潜力的十大风景名胜区”，五大连池市获得“中国十大休闲城市”称号。

——重点项目取得积极进展。续建和新开工重点项目28项，完成投资24.5亿元，增长47.5%。三道湾子岩金矿、茅宝沟铁钼矿、兴边煤矿、盛泰硅业、联凯大豆一期等项目建成投产，嫩江至多宝山220千伏输电线路投入使用。大多宝山铜矿、翠宏山铁多金属矿、关鸟河水泥厂、粉煤灰综合利用一期工程、象山水库除险加固、金湾水电站、海信热电厂、大黑河岛自由贸易广场以及黑呼公路黑河段、嫩呼公路嫩江至多宝山段、111国道嫩江段升级改造等项目进展顺利，绝大多数完成了年度建设目标。东安岩金矿、海澜热电厂、国家大型商品粮基地、嫩黑公路等项目前期工作进度加快。三吉屯120万千瓦煤电一体化项目与国电集团签订协议，前期工作已经启动。黑河俄电加工区和对俄进出口加工区、北安工业园区、五大连池风景区综合开发区等园区基础设施日臻完善。这些都使我市经济发展后劲明显增强。

——城乡人居环境更加优化。全市城市基础设施建设投入3亿元，重点城镇基础设施不断完善。聘请国内著名城市规划专家，对城市建设进行了策划。市区完成投资1.5亿元，中央街东段道路拓宽、龙滨路和龙源路人行道铺装、住宅小区改造等工程竣工，通江路公共建筑物实行亮化，城市品位进一步提高。西部棚户区改造加快推进，完成动迁852户，其中第一批442户居民已回迁入住。东部棚户区改造、污水处理和医疗垃圾处理工程前期工作基本完成。北安、嫩江、逊克道路改造和五大连池、孙吴城区硬化、亮化、绿化项目加快建设。全市新建住宅112万平方米，人均住房建筑面积23平方米，高层建筑增势加快，节能建筑全面铺开。农村住房砖瓦化率61%，自来水普及率63%。严格监控企业污染物排放，俄电加工区企业排污得到有效治理，城市空气质量达到二级天数360天。生态市建设进一步加强，北安市通过国家级生态示范区验收。

——社会事业全面协调发展。实施省级以上科技项目14项，省级硅基材料工程技术研究中心、对俄科技交流与合作中心正式在黑河成立。引进人才智力取得新成果，招聘重点院校毕业生108名，其中硕士研究生11名。“两基”工作通过国家验收，小学和初中辍学率分别控制在0.02%和0.67%。成立市属黑河小学，与黑河三中合并组建九年一贯制学校，易地新建的黑河中学主体工程完工。农村义务教育经费保障机制改革全面实施。市第一医院门诊大楼开工建设，改扩建8个乡镇卫生院，新型农村合作医疗农民参合率达到93.2%。群众文化体育活动蓬勃开展，获得省级以上体育比赛奖牌69枚，市青少年活动中心、群众文化活动中心和老干部活动中心主体工程完工，市广播电视中心前期工作基本完成。旅俄华侨纪念馆建成开馆，黑龙江知青馆主体工程完工。市区12个社区办公用房全部建成。人口自然增长率3.16‰。黑河荣获全国双拥模范城称号。实施“春蕾计划”，资助贫困女童643名。档案管理信息化建设取得新进展。民族宗教、侨务、老龄、人防、地方史志等事业全面发展。

——就业和社会保障加快推进。全市新增城镇就业1.7万人，登记失业率4%，1984户零就业家庭全部实现就业。黑河对俄劳务输出实训基地主体工程完工。社会保险覆盖面不断扩大，各类保险金发放率100%。城市低保月人均补差由84元提高到99元，农村低保年人均补差由351元提高到400元，均达到省标准。优抚对象抚恤补助标准和医疗保障水平进一步提高。各县（市）区新建6所中心敬老院全部投入使用。廉租住房工作全面启动，1714户最低收入家庭受益。

——安全稳定形势总体趋好。信访工作不断加强，矛盾纠纷排查工作扎实推进，一批重大疑难信访问题得到有效化解。安全生产形势稳定好转，煤矿等重点领域隐患排查和专项整治扎实有效，全市共发生各类事故297起，下降21.6%，死亡人数和经济损失分别下降19.7%和11.1%。森林防火体系不断健全，基础设施有所改善，新增400名森警和1000人地方扑火专业队作用突出，综合防控能力明显提高，连续三个防期没有发生重特大森林火灾。平安黑河建设稳步推进，市区重点地段实行24小时动态巡逻，治安防控网络更加严密。边境管理得到加强，边境秩序安全稳定。

——政府自身建设进一步加强。自觉接受市委领导和人大、政协监督，出台了《主动接受人民政协民主监督的意见》，办理人大代表、政协委员建议和提案105件，办结率100%。推进依法行政工作，进一步规范行政权力和行政行为，行政执法权力和其他行政权力分别削减21%和12%。对全市重大工程、土地出让等事项实行公开，群众知情权得到有效保障。编制应急预案50个，应急救援体系初步建立。公务员教育和目标管理、政务督查得到加强，工作推进落实能力有所提高。党风廉政建设工作取得进展。

各位代表，过去的一年，是新一届政府的开局之年，我们在新起点上迈出了新步伐。这些成绩的取得，是省委省政府正确领导、亲切关怀的结果，是市委统揽全局、科学决策的结果，是人大依法监督、大力支持的结果，是政协民主监督、参政议政的结果，是驻黑中省直单位和人民解放军、武警官兵以及全市公安政法干警密切配合、有力保障的结果，是全市人民团结一致、奋勇拼搏的结果。在此，我代表市政府表示崇高敬意和衷心感谢！

在肯定成绩的同时，我们也清醒地看到，全市经济社会发展中还存在一些不容忽视的困难和问题。主要表现在：发展速度低于全省平均水平，加快发展压力大；农业基础设施落后，抵御自然灾害能力弱；工业总量不足，新型工业化任重道远；外经贸运行质量有待提高，贸易与产业关联度低；域外人口流入量小，消费拉动增长不快；财政刚性支出因素增多，收支矛盾依然突出；金融市场还不活跃，对地方支持作用仍然较弱；物价涨幅较大，城乡居民收入较低，群众关注的一些热点难点问题解决得还不够好，等等。政府工作也存在缺点和不足，主要是部门少数同志思想不够解放，工作标准低，办事效率不高。这些问题我们一定要高度重视，认真对待，在今后工作中着力加以解决。

二、促进经济又好又快发展

2008年是我国改革开放第三十年，还将举办北京奥运会，同时也是我们深入贯彻落实党的十七大精神、努力实现“十一五”规划的重要一年，做好今年工作，意义十分重大。我们要以邓小平理论和“三个代表”重要思想为指导，坚持科学发展观，认真贯彻落实党的十七大和中央经济工作会议精神，正确理解和把握中央宏观调控政策，围绕打造北疆口岸名城、建设小康黑河、和谐黑河的目标，加快资源产业开发，扩张经济总量，增加投资和消费，夯实发展基础，转变发展方式，加快发展步伐，着力保障和改善民生，实现经济与社会同步推进，人与自然和谐发展。主要预期目标是，地区生产总值增长10%，城镇固定资产投资增长20%，社会消费品零售总额增长12%，对外贸易进出口总值增长20%，全口径财政收入增长11%，城镇居民人均可支配收入增长9%，农民人均纯收入增长7%，万元地区生产总值能耗降低3.6%，化学需氧量保持不上升，二氧化硫排放量下降0.5%。实现今年经济发展目标，我们要统筹全局、把握重点，集中精力抓好以下六个方面工作。

（一）加快产业发育进程，努力转变经济发展方式

产业发育是实现黑河经济又好又快发展的重要支撑。必须立足区位、资源和生态优势，紧紧围绕园区建设，大力培育具有核心竞争力的优势产业集群。同时，发育产业绝不能以浪费资源、牺牲环境为代价，必须提高科技含量、降低资源能源消耗、严控环境污染，保持生态良好的后发优势，实现可持续发展。全市规模以上工业增加值增长15%。强力推进优势产业。我市具有丰富的矿产资源，巨大的火电、水电、风电潜力，得天独厚的口岸条件，广袤的土地、森林，这是优势所在。我们要紧紧围绕这些优势，强力做大产业规模。要重点发展工业硅、碳化硼下游产品，放大俄电优势。要抓好煤炭和火电、水电、风电开发。关鸟河水泥厂一期工程和东兴、金三角等煤矿投产，力争达产达效。加快建设大多宝山铜矿和翠宏山铁多金属矿。争取开工争光岩金矿、庆联石化公司30万吨电石一期工程、联凯大豆加工二期工程、海澜和孙吴热电厂等项目。积极推进五大连池矿泉水生产扩能改造，统筹五大连池市与景区协调发展。抓好东安岩金矿、合盛集团年产20万吨有机硅、鑫恒集团年产50万吨铝综合加工等项目前期。配合国电集团全力做好三吉屯120万千瓦煤电一体化项目前期工作。阿州—黑河油品储运和24万吨磷酸二铵项目取得实质性进展。积极引进出口加工企业。在抓好产业项目的同时，深入研究我市功能分区定位，争取将重点产业带和园区纳入省主体功能区规划。继续完善俄电加工区、对俄进出口加工区等园区基础设施，加快推进爱辉、北安、嫩江工业园区项目建设，特别是北安、五大连池要克服资源短缺的劣势，发挥中心区位的优势，大力引进多门类的发展项目。支持省电力公司开展对俄购电业务，扩大俄电供应范围。加快科技创新步伐。抓好工业硅节能环保冶炼技术应用、碳化硼深加工等省级火炬计划项目，开发硅基新材料系列产品。加强与国内外科研机构协作，加快成果转化，争取辟建国家级技术创新基地。扶强省级高新技术企业和民营科技企业，利用信息技术提升传统产业。大力加强生态建设。发展循环经济，以俄电加工区、多宝山矿业开发区、三吉屯煤电经济区为重点，提高资源综合利用率。加大节能减排力度，抓好黑河热电厂等重点用能大户节能降耗。争取粉煤灰综合利用项目一期工程完工，为建筑企业提供接续建材。按照规划，继续推进生态市建设。严格执行环保法规，新建项目严把环保审批关，建成项目排污必须达标。扩大集中供热，改善空气质量。加强对界江、森林、草原、湿地的生态保护，饮用水源地水质达标率100%。强化经济运行监控和推进。加强监测分析和预警工作，密切跟踪原材料、能源、粮食等市场价格动态，做好调查研究，及时发现苗头性、倾向性问题，积极采取应对措施。加大煤电油运协调力度，保证供给，提高经济运行质量和效益。今年政府组成人员都要承担项目推进责任，做到全市上下合力抓经济。

（二）进一步扩大对外开放，提升外经贸运行质量

国家东北振兴与俄远东开发战略同步实施，黑河作为两大板块重要交汇点，必须放大窗口、平台、通道和基地作用，加快对俄经贸科技合作战略升级，促进中俄地区间合作。继续推进“两国一城”建设。进一步完善两地政府间定期会晤机制、部门工作协调机制，建立更加密切的合作关系。围绕“两国一城”建设各项任务，与俄方共同策划、开展资源开发、经贸、科技、教育、文体、卫生等领域的交流合作。引进扶强外经贸主体。落实外贸奖励政策和鼓励进口优惠政策，着力吸引进口和有自营进出口权的大户。对重点企业实行跟踪服务，帮助协调解决融资、出口退税、口岸运输等困难。及时掌握国内外最新经贸政策和信息，指导企业积极应对，提高抗风险能力。改善贸易结构。进一步调整贸易方式，大力发展边境小额贸易和边民互市贸易，提高一般贸易中的自营份额，增强对外贸易稳定性。调整进出口商品结构，扩大化工、木材、矿产品、机电产品等大宗商品进口，进口总值增长30%；增加汽车、工程和农用机械、电子产品等高附加值商品出口，提高农副产品、水泥等地方产品出口比重，提升贸易对地方经济贡献率。支持企业走出去在俄创业，推进以单纯的贸易为主向贸工、贸农、贸技相结合转变。营造优良口岸环境。充分发挥黑河新货运口岸作用，提高通关效率，降低通关成本，过货量增长30%。加强口岸客货运输协调，解决俄方通关瓶颈问题，力争实现双方货检现场每周七天工作制。落实《关于保障俄罗斯公民相关权益的意见》，组建服务机构，在公共场所设置俄文标识，为俄罗斯公民在互贸区投资消费，创造安全公平便利的环境。做好招商引资工作。围绕矿产资源、进出口加工、俄电、农业、旅游、物流、城市功能等领域，进一步完善项目储备库。要转变招商方式，以小型、对口登门、经常性招商为主。要面向国内国际五百强企业招商，面向与我市开发导向相一致的企业招商，面向有对俄出口商品的强势企业招商。力争招商引资到位资金37亿元，增长30%。

（三）积极发展现代农业，促进农民持续增收

解决好“三农”问题，事关经济发展和社会稳定全局。必须坚持城乡统筹，深入推进新农村建设，用现代发展理念引领农业，用现代产业体系提升农业，促进农业持续发展和农民持续增收。农业增加值增长4%以上。深入推进场县共建。按照区域一体化思路，全面推广嫩江与九三分局共建经验，积极探索其他县（市）区共建模式，强化市场主导，拓宽领域，提高层次。扩大农机跨区作业规模，加快推广农场模式化栽培，建设500万亩示范区。共同扶壮北大荒麦业、九三油脂等10户重点加工企业，合作共建嫩江科洛、北安通北等10个小城镇，整合公共资源，促进共同发展。扶持壮大畜牧养殖业。落实优惠政策，加大扶持力度，促进产业发展。调整优化布局，建设北安、五大连池、嫩江奶牛、肉牛、肉羊及北部县（区）大鹅养殖基地。扩大兔、狐、貉等特色养殖规模。抓好配套体系建设和疫病防治。畜牧业增加值增长10%。加快农业机械化进程。按照省政府批准的三年组建规划，组建农机合作社50个。完善机制，加强管理，实现农机合作社高效率运营。坚持农机农艺相结合，推广农机标准化作业，全面推开“五秋”整地，总量达到应整地面积的三分之一以上，努力提高单位资源产出率，缩小与农场单产差距。以农机化带动农业生产规模化、标准化和科学化，提高农业科技贡献率。加快林权改革和林产经济发展。推进爱辉区林权改革试点，积极探索其他县（市）改革思路，搞活林业经营体制。大力发展林下经济，扩大北药生产基地规模，开发生态旅游产品，促进林业产业发展，林业产业产值增长27%。加强对俄合作和产业开发，提高沙棘、花楸等树种品质。制定速生丰产林整地还林规划，为还林奠定基础，充分发挥林业效益。提高产业化经营水平。继续推进现代农业产业带建设，启动国家大型商品粮基地项目。按照产业化经营思路，依托嫩江南华糖业公司、孙吴联凯油脂集团及北安完达山乳业、九三大洲肉业等龙头企业，发展麦、豆、薯、菜、麻及奶、肉等精深加工业。合理规划产业布局，扩大生产基地规模。完善企业、基地、农户间利益联结机制，促进产销对接。发展农村合作经济组织。贯彻落实《农民专业合作经济组织法》，按照专业化、市场化原则，推进农机服务组织体系建设，加快推广股份合作、托管经营、统种统收等模式，促进土地集约化规模经营。发展农村专业协会、中介服务、市场流通等专业组织，壮大农村经纪人队伍，提高农民组织化程度。促进农村劳动力转移。落实新型农民培训计划，提高农民综合素质和劳动技能。搞好指导联络服务，组织农民外出打工，保护农民工合法权益。坚持创业带动就业，积极发展劳动密集型产业，促进就地转移。扩大境外土地开发规模，带动劳务输出。转移农村劳动力17万人。强化农业基础设施建设。加强农田水利建设，完成爱辉卧牛湖、嫩江青年、北安石远等水库除险加固工程，搞好小型水利建设。新建农村人畜饮水工程20处，治理水土流失10.5万亩。落实责任，加强耕地保护。建设水源和人工影响天气工程，提高抗御自然灾害的综合能力。加快小城镇、中心村基础设施建设，提高公共服务水平，改善农村生产生活条件。

（四）发挥投资消费拉动作用，增强经济发展动力

投资和消费是加快经济发展的主要驱动力，就黑河而言，投资尤为重要。必须充分认识当前黑河发展的阶段性特征，坚定不移实施大项目牵动战略，不断扩大投资规模。促进固定资产投资增长。续建和新开工重点项目37项，安排投资45.9亿元。除工业、城建等项目外，继续加大交通、水利等基础设施投入力度。黑呼公路黑河段、嫩呼公路嫩江至多宝山段竣工通车，111国道嫩江段、加黑公路黑河至罕达气段路基和桥涵工程完工，升级改造嫩黑公路、黑嘉公路逊克段，建设1110公里农村公路，实现全部乡镇和65%的行政村通白色路面。协调俄方完成国内审批程序，力促黑龙江大桥早日开工。加快金湾水电站和黑龙江黑河城区段堤防建设，推进二道坎水库和新鄂、乌二水电站前期工作。支持国家电网公司500千伏中俄直流背靠背输电项目建设，配合做好黑河至多宝山220千伏输电项目前期工作。努力扩大城乡消费需求。推进大型物流配送、展销、连锁超市、专业批发市场建设，发挥对区域消费的带动作用。大黑河岛自由贸易广场投入运营，国际物流区对外招商，北安建材大市场、北方物流配送中心和嫩江新型商务服务中心开工建设。继续实施“万村千乡”市场工程，全市乡村覆盖率达到98%。鼓励城乡居民购置住房、汽车和中高档消费品，推动文化、教育、健身等新兴消费。提升壮大旅游经济。加快推进旅游带建设，加大“完美旅游线路” 开发力度，实施黑河市区与景区互动，放大五大连池景区品牌效应，接待游客突破100万人次，力争早日成为国家5A级景区。促进孙吴二战遗迹旅游区提档升级。加快市区旅游开发，推进卧牛湖景区、中俄民族风情园、瑷珲历史文化旅游区等重点景区（点）建设。打造旅游景观，建设北方明珠塔。积极争取增加进哈列车，开通北京航线，争取黑河尽快恢复异地办照。全市接待国内外旅游者138万人次，其中边境游47万人次，均增长10%。加快发展试车经济。千方百计争取国家行业部门的政策、资金支持，力争国家级汽车低温技术测试中心在黑河落地。加强与韩、日、德等国汽车企业及北欧、新西兰试验场合作。继续吸引国内外汽车生产企业、研发机构投资建场，完善服务体系，把我市建成以大型综合试车场为核心、以企业自建零部件试验场为辅助的国际一流寒区试车基地。积极培育度假经济。突出夏季避暑养生、冬季体验冰雪风情等特色主题，积极打造“候鸟式、公寓式”居住环境，吸引俄罗斯公民和南方居民、客商到黑河休闲度假和投资置业，聚集人气，拉动消费。针对俄居民消费需求，规划建设娱乐、商务等功能区，为俄居民入境休闲度假、观光购物创造条件。大力发展会议经济。依托“两国一城”城市品牌和区位优势，捕捉信息，推介优势，积极申办国际会议和高层论坛。加强信息沟通，强化对接协调，争取承办国家和省级行业会议。充分发挥行业协会、专业学会及企业集团自身优势，创造条件举办各类理论研讨和商务会议。完善硬件设施，鼓励创办会议公司等中介组织，全面提高会议服务水平。不断扩大对外宣传。充分利用国内相应媒体，积极参加旅游等推介活动，提高知名度，增强吸引力。在俄远东地区举办“黑河宣传周”活动，聚集境外消费群体。今年政府将安排专款，推进这项工作。

（五）扎实开展创建活动，努力建设宜居城市

城市的规划建设管理群众关注度高，与群众生活质量息息相关。各县（市）区要依据城市规划，加快完善县城和城镇功能，增强综合承载力，满足发展需要；市区要把握临江、口岸特点，围绕宜居城市目标，以创建活动为载体，不断提升城市品位。推进城市创建活动。巩固全国双拥模范城，争创中国优秀旅游城及全国环保模范城、卫生城、园林城、平安城、文明城，打造北方文化名城，与经济社会发展统一推进，调动市民参与热情，提高市民文明素质，改善居民生活环境。完善城市规划体系。调整城市发展战略，做到“东扩、西改、南调、北优”。依据城市定位，突出城市特色，进一步完善城区建设总体规划，编制大黑河岛整体保护利用规划及城市功能分区控详规划。抓好城中村改造，做好城区旅游景观、绿地系统、环境保护及棚户区改造等专项规划修编工作。加大行政监管力度，严格执行既定规划，坚决制止违规行为，维护规划权威性和严肃性。成立专家委员会，加强对新建楼房立面的把关审查，保证造型美观和个性鲜明。抓好重点工程建设。开工市区污水和医疗垃圾处理项目，启用生活垃圾处理工程。完善城区给排水设施，综合治理南大沟，消除市区水患。续建南出口路拓宽工程，改造环城路西段，筹建改造大岛桥。抓好黑龙江公园、中央街东段及城区部分节点绿化、亮化。全面启动市区主要街路立面粉饰工程，装饰改造黑龙江护栏。加快改造西部棚户区，完善道路、供热、电力、给排水等配套设施。启动东部棚户区回迁楼建设。开工嫩江和五大连池污水处理工程，新建北安商业街，扩建逊克江畔公园，拓宽孙吴中央街、红旗街。提高城市管理水平。加大投入，落实责任，提高标准，文明执法。出台并严格执行道路、市容、绿化和广告等20个管理办法，全面推行门前卫生、市容秩序、立面整洁、绿化管护、文明行为的“五包”责任制，推进城市精细化管理。城区户外广告、绿地管护、道路养护等全面推行招标管理。加强物业管理，维护居民合法权益。加大经营性用地“招拍挂”出让力度，提高土地收益。

（六）做好财税金融工作，强化公共保障能力

财税金融工作事关经济发展和社会建设。必须进一步完善公共财政体系，发挥政策、资金导向作用。大力培植新兴财源。巩固基础财源，培植比较效益高的新兴、后续财源，争取更多项目纳入省重点财源建设扶持范围。加强协调指导，同步推进县（市）区财源建设。加大财税征管力度。强化重点税源监控，深入开展煤炭、建筑等重点行业税收专项征缴活动，依法征收，应收尽收。完善税源动态管理机制，抓好非税收入和零散税种征收。加大征管稽查力度，严厉打击偷逃骗税，严肃查处违规行为。提高财政保障能力。多方筹措资金，保障公共支出。深入研究有关政策、资金渠道，加大向上争取力度。集中财力支持大项目前期、新农村建设、公益事业和民生工程。市直按月人均标准300元兑现2007年津补贴政策，行政事业单位职工住房公积金财政匹配比例由5%提高到8%。强化重点项目、重点资金审计监督，提高财政资金使用绩效。深化财政体制改革。抓住我市被省财政厅列为改革试点契机，深入推进财政支出管理、财政支出绩效评价、部门预算定员定额等试点改革，健全政府性投资重点工程规划设计、重点项目招投标及资金投入评审论证体系。搞好国有资产整合、置换和变现，提高利用效益。严格执行《政府采购法》，深化政府采购制度改革，扩大采购范围，规范采购行为。改善金融生态环境。加强和改进政府对金融机构的服务，积极帮助解决实际困难和问题。建立有效的金融机构支持地方经济发展的激励机制，鼓励金融机构加大对地方经济的信贷投入，增强拉动作用。充分发挥金融办的桥梁和纽带作用，搭建政府部门、银行、企业间信息交流平台。推进信用体系建设，建立企业信用评价、监督、管理等信用制度，营造良好的社会信用环境。

三、加快推进和谐黑河建设

社会建设与人民幸福息息相关。要按照党的十七大要求，在促进经济发展的同时，更加注重社会建设，保障和改善民生，保持社会安全稳定，努力建设和谐黑河。

（一）协调发展社会事业，着力保障和改善民生

民生问题是政府工作的出发点和落脚点。必须坚持以人为本，按照“学有所教、劳有所得、病有所医、老有所养、住有所居”要求，让群众共享发展成果。大力发展教育事业。重视学前教育，关心特殊教育，抓好义务教育，加快普及高中阶段教育，大力发展职业教育，促进教育协调发展。小学和初中辍学率分别控制在1%和3%以下，高中阶段毛入学率达到55%以上。突出办学特色，抓好逸夫中学、黑河三中规范化管理，黑河中学要全面提高教学质量，争创名牌学校。加强教育研究，深化课程改革。继续实施农村义务教育保障机制，保证困难家庭、进城务工人员子女接受义务教育。依托黑河学院，开展市院共建，鼓励院企合作，促进教育资源共享。积极扩大就业再就业。深入贯彻《劳动合同法》和《就业促进法》，落实就业政策，扩大就业规模，新增就业1.4万人，城镇失业率控制在4.5%以下。发挥教育、培训、实训基地作用，紧紧围绕大项目建设，提前实施订单培训，有针对性地培养和储备一批成熟的产业工人，提高供需对接度。健全就业援助制度，实行就业实名制管理。开展鼓励创造就业岗位和自主创业的“双创”活动，对聘用零就业家庭成员、黑河籍高校毕业生的企业和自主创业的黑河市国有企业下岗失业人员，给予一定补贴，确保零就业家庭出现一户，解决一户，帮助下岗失业人员、高校毕业生充分就业。不断提高医疗卫生服务水平。坚持公共医疗卫生公益性质，建立基本医疗卫生制度。加强城市社区卫生服务体系和县、乡、村三级卫生服务网络建设。巩固新型农村合作医疗成果，落实国家提高补助标准政策，进一步减轻农民看病负担。推进市第一人民医院门诊大楼建设，抓好市中医院搬迁扩建前期工作，加强乡镇卫生院建设，培育特色医院和诊疗专科。加强监管，切实解决群众看病贵问题。完善重大疾病防控体系，提高突发公共卫生事件应急处理能力和医疗救治能力。坚持计划生育基本国策，提高人口素质，稳定低生育水平，人口自然增长率控制在3.9‰以内。大力加强市场监管。整顿和规范市场经济秩序，加强食品、药品监管，严厉打击商业欺诈。健全市场价格监测预警机制，依法打击价格违法违规行为，抑制物价过快上涨。发挥价格调节基金作用，切实保护低收入群体利益。全面做好社会保障工作。加大财政投入，加强基金征缴，扩大工伤、失业、生育保险覆盖面，确保高风险行业职工特别是农民工全部纳入工伤保险范围。全面推行事业单位基本养老保险制度，启动城镇居民医疗保险试点，做好灵活就业人员试点准备工作。提高城乡困难家庭医疗救治水平。进一步完善城乡社会救助体系，提高城乡最低生活保障额度，实现应保尽保和资金足额发放。建立政府主导、社会参与的养老机制，确保孤寡老人老有所养。加强城市住房保障体系建设，建立经济适用住房和廉租住房保障机制，解决城市低收入家庭住房困难问题。统筹发展其他各项事业。大力发展文化事业和文化产业，加大基础设施投入，推进和谐文化建设。加强与俄艺术团体的合作交流。继续开展广场演出和农村大院活动，丰富群众文化生活。以迎奥运为契机，开展全民健身活动，提高竞技体育水平。发展广播电视事业，开工建设广电中心，加快有线电视数字化。大力发展社区服务业，启动爱辉、嫩江10乡10村社区试点。做好优抚安置双拥工作，发展残疾人事业。加快老区开发，促进老区发展。做好妇女儿童工作，全面落实新一轮妇女儿童规划目标。加强档案管理维护，加快数字化档案馆建设。同时，进一步做好民族宗教、侨务、老龄、人防、地方史志等工作。

（二）努力减少不和谐因素，维护社会安全稳定

经济社会发展需要以稳定为基础。必须努力维护社会安全稳定，为又好又快发展提供有力保障。全力抓好森林防火工作。巩固森防成果，加强火源管理，抓好防火道路、通讯网络、阻隔设施及指挥体系、扑救队伍建设，提高预警和扑救能力，确保不发生重特大火灾。改造扩建黑河航站，启动建设卧都河、白云移动航站，争取逊克、孙吴移动航站尽快立项。完善森林管护制度，全面推广爱辉区森防托管责任制做法，扩大托管规模，力争达到750万亩。同时做好防汛工作，全面落实防汛措施和责任制，确保全市各类水库和江河安全度汛。强化安全生产监管。坚持安全发展，严格执行安全生产责任制，加强对煤矿、交通、消防、民爆、水运、工程建筑、公共场所等重点行业和领域的隐患排查和专项整治，杜绝重特大安全事故，保护人民生命财产安全。加大信访工作力度。完善长效机制，畅通群众信访渠道，强化责任落实，维护群众合法权益。做好矛盾纠纷排查调处工作，妥善处理突发性群体事件。建设接访大厅，规范信访秩序。加快推进平安黑河建设。强化社会治安综合治理，依法防范和严厉打击各类刑事犯罪和经济犯罪。实施全警巡逻防控工作机制，建设治安防范视频监控系统和社区警务工作室，健全防控体系。加强边境管理，维护边境安全稳定。深入开展“五五”普法教育，实施依法治市规划。切实做好应急救援工作。健全各级应急预案和救援机制，完善自然灾害应急救助和转移安置体系，确保救援快速、处置有效。积极发展慈善公益组织，广泛开展社会捐助活动，解决受灾群众生产生活问题。

四、努力建设服务型政府

全面贯彻落实科学发展观，对各级政府提出了更新更高的要求。必须加快职能转变，创新运行机制，提高公共服务水平。把握服务定位。强化公仆意识，牢记为民宗旨，正确处理管理与服务的关系，切实为群众办实事。坚决贯彻市委决策，依法接受市人大及其常委会监督，积极支持人民政协履行职能，认真办理人大代表建议和政协委员提案，主动听取各民主党派、工商联和各人民团体的意见和建议。落实服务责任。坚持依法行政，实施行政执法责任制和行政执法绩效评估，减少执法层级，规范执法程序，坚决防止行政不作为和乱作为。加强行政审批工作监督管理，出台机关工作人员绩效考核办法，实施行政问责制，确保政府权力规范运行。严格执行督办检查规定，工作一旦决定，言出必践。健全服务机制。完善政府议事规则、重大事项集体决策、专家咨询论证和大额资金使用制度。继续推行政务公开，畅通了解民情、集中民智的渠道，对与人民群众利益密切相关的重大事项，实行社会公示、听证，保证群众的知情权和参与权。建立大项目专项推进服务机制，积极帮助外来企业解决问题。办好市长信箱和市长热线，加强社区服务网络建设，为群众提供便捷服务。提高服务效能。加强学习和调查研究，吃透上情，提高对上级政策的领悟能力；掌握下情，增强解决实际问题的针对性。鼓励公务员从实际出发，创造性地开展工作；倡导勤奋敬业，坚持高标准严要求，切实提高服务效率和质量。推进事业单位机构改革，由办事业向管事业转变。加快电子政务建设，整合政府网络资源，实现资源共享。强化服务保障。认真执行廉政建设规定，健全建筑工程招投标、经营性土地使用权出让、产权交易、政府采购等监督约束机制。落实“入企报告制”和“责任追究制”，营造良好发展环境。完善政府诚信机制，努力建设诚信政府。强化群众和媒体监督，维护社会公平正义。

各位代表，2008年任务繁重而艰巨，我们将认真履行职责，全力做好政府各项工作。在统筹兼顾的同时，突出重点，集中精力，积极培育旅游经济、试车经济、度假经济、会议经济等4种新型经济，推进18项重点工程建设，实施12项改善民生行动。在重点工程建设方面，加快推进三吉屯120千瓦煤电一体化、金湾水电站、阿州—黑河油品储运、黑龙江大桥、关鸟河水泥厂、大多宝山铜矿、翠宏山铁多金属矿、争光岩金矿、海澜热电厂、庆联公司电石开发、黑河至呼玛公路、嫩江至多宝山公路、黑河至嫩江公路、五大连池矿泉水扩能改造、国家大型商品粮基地、俄电加工区、北安工业园区和城市建设等项目。在改善民生行动方面，易地新建黑河一中投入使用，着力提高教学质量；开展“双创”活动，新增就业1.4万人；启动城镇居民医疗保险试点，巩固新型农村合作医疗，财政补贴标准提高到80元；建设第一医院门诊大楼，改善群众就医条件；完善城乡最低生活保障制度，实现应保尽保和资金足额发放；开工建设市区污水和医疗垃圾处理工程，生活垃圾处理工程投入使用；综合治理南大沟，消除市区水患；拓宽南出口路，改造环城西路，创造良好的交通条件；建立经济适用住房和廉租住房保障机制，解决低收入家庭住房困难；继续改造西部棚户区，建设东部棚户区回迁楼；改善供热条件，市区新增供热面积200万平方米；打造“平安黑河”，建设治安防范视频监控系统。

各位代表，黑河正处在夯实基础、力促发展的关键时期，我们深感责任重大。让我们在市委的正确领导下，贯彻落实科学发展观，解放思想、开拓创新、加压奋进、埋头苦干，为实现经济社会又好又快发展，推进小康黑河、和谐黑河建设而努力奋斗！
